# Effects of ovarian stimulation on embryo euploidy: an analysis of 12 874 oocytes and 3106 blastocysts in cycles with preimplantation genetic testing for monogenic disorders

**DOI:** 10.1093/hropen/hoae054

**Published:** 2024-10-03

**Authors:** Congcong Ma, Xiaoyu Long, Liying Yan, Xiaohui Zhu, Lixue Chen, Rong Li, Ying Wang, Jie Qiao

**Affiliations:** Department of Obstetrics and Gynecology, Center for Reproductive Medicine, Peking University Third Hospital, Beijing, China; National Clinical Research Center for Obstetrics and Gynecology, Beijing, China; Key Laboratory of Assisted Reproduction (Peking University), Ministry of Education, Beijing, China; Beijing Key Laboratory of Reproductive Endocrinology and Assisted Reproductive Technology, Beijing, China; Department of Obstetrics and Gynecology, Center for Reproductive Medicine, Peking University Third Hospital, Beijing, China; National Clinical Research Center for Obstetrics and Gynecology, Beijing, China; Key Laboratory of Assisted Reproduction (Peking University), Ministry of Education, Beijing, China; Beijing Key Laboratory of Reproductive Endocrinology and Assisted Reproductive Technology, Beijing, China; Department of Obstetrics and Gynecology, Center for Reproductive Medicine, Peking University Third Hospital, Beijing, China; National Clinical Research Center for Obstetrics and Gynecology, Beijing, China; Key Laboratory of Assisted Reproduction (Peking University), Ministry of Education, Beijing, China; Beijing Key Laboratory of Reproductive Endocrinology and Assisted Reproductive Technology, Beijing, China; Department of Obstetrics and Gynecology, Center for Reproductive Medicine, Peking University Third Hospital, Beijing, China; National Clinical Research Center for Obstetrics and Gynecology, Beijing, China; Key Laboratory of Assisted Reproduction (Peking University), Ministry of Education, Beijing, China; Beijing Key Laboratory of Reproductive Endocrinology and Assisted Reproductive Technology, Beijing, China; Department of Obstetrics and Gynecology, Center for Reproductive Medicine, Peking University Third Hospital, Beijing, China; National Clinical Research Center for Obstetrics and Gynecology, Beijing, China; Key Laboratory of Assisted Reproduction (Peking University), Ministry of Education, Beijing, China; Beijing Key Laboratory of Reproductive Endocrinology and Assisted Reproductive Technology, Beijing, China; Department of Obstetrics and Gynecology, Center for Reproductive Medicine, Peking University Third Hospital, Beijing, China; National Clinical Research Center for Obstetrics and Gynecology, Beijing, China; Key Laboratory of Assisted Reproduction (Peking University), Ministry of Education, Beijing, China; Beijing Key Laboratory of Reproductive Endocrinology and Assisted Reproductive Technology, Beijing, China; Department of Obstetrics and Gynecology, Center for Reproductive Medicine, Peking University Third Hospital, Beijing, China; National Clinical Research Center for Obstetrics and Gynecology, Beijing, China; Key Laboratory of Assisted Reproduction (Peking University), Ministry of Education, Beijing, China; Beijing Key Laboratory of Reproductive Endocrinology and Assisted Reproductive Technology, Beijing, China; Department of Obstetrics and Gynecology, Center for Reproductive Medicine, Peking University Third Hospital, Beijing, China; National Clinical Research Center for Obstetrics and Gynecology, Beijing, China; Key Laboratory of Assisted Reproduction (Peking University), Ministry of Education, Beijing, China; Beijing Key Laboratory of Reproductive Endocrinology and Assisted Reproductive Technology, Beijing, China; Beijing Advanced Innovation Center for Genomics, Beijing, China; Peking-Tsinghua Center for Life Sciences, Peking University, Beijing, China

**Keywords:** ovarian stimulation, aneuploidy, preimplantation genetic testing for monogenic disorders, blastulation rate, gonadotropins

## Abstract

**STUDY QUESTION:**

Does ovarian stimulation and the ovarian response affect embryo euploidy?

**SUMMARY ANSWER:**

Ovarian stimulation and the ovarian response in women undergoing preimplantation genetic testing for monogenic disorders (PGT-M) cycles did not affect the rates of blastocyst euploidy.

**WHAT IS KNOWN ALREADY:**

Whether or not ovarian stimulation in IVF–embryo transfer has potential effects on embryo euploidy is controversial among studies for several reasons: (i) heterogeneity of the study populations, (ii) biopsies being performed at different stages of embryo development and (iii) evolution of the platforms utilized for ploidy assessment. Patients who undergo PGT-M cycles typically have no additional risks of aneuploidy, providing an ideal study population for exploring this issue.

**STUDY DESIGN, SIZE, DURATION:**

A retrospective cohort study including embryos undergoing PGT-M was conducted at a single academically affiliated fertility clinic between June 2014 and July 2021.

**PARTICIPANTS/MATERIALS, SETTING, METHODS:**

A total of 617 women with 867 PGT-M cycles involving 12 874 retrieved oocytes and 3106 trophectoderm biopsies of blastocysts were included. The primary outcome of the study was median euploidy rate, which was calculated by dividing the number of euploid blastocysts by the total number of biopsied blastocysts for each cycle. Secondary outcomes included the median normal fertilization rate (two-pronuclear (2PN) embryos/metaphase II oocytes) and median blastulation rate (blastocyst numbers/2PN embryos).

**MAIN RESULTS AND THE ROLE OF CHANCE:**

Comparable euploidy rates and fertilization rates were observed across all age groups, regardless of variations in ovarian stimulation protocols, gonadotropin dosages (both the starting and total dosages), stimulation durations, the inclusion of human menopausal gonadotrophin supplementation, or the number of oocytes retrieved (all *P* > 0.05). Blastulation rates declined with increasing starting doses of gonadotropins in women aged 31–34 years old (*P* = 0.005) but increased with increasing gonadotrophin starting doses in women aged 35–37 years old (*P* = 0.017). In women aged 31–34, 35–37, and 38–40 years old, blastulation rates were significantly reduced with increases in the number of oocytes retrieved (*P* = 0.001, <0.001, and 0.012, respectively).

**LIMITATIONS, REASONS FOR CAUTION:**

Limitations include the study’s retrospective nature and the relatively small number of patients of advanced age, especially patients older than 40 years old, leading to quite low statistical power. Second, as we considered euploidy rates as outcome measures, we did not analyze the effects of ovarian stimulation on uniform aneuploidy and mosaicism, respectively. Finally, we did not consider the effects of paternal characteristics on embryo euploidy status due to the fact that blastocyst aneuploidy primarily originates from maternal meiosis. However, sperm factors might have an effect on embryo development and the blastulation rate, and therefore also the number of blastocysts analyzed. The exclusion of patients with severe teratozoospermia and the fact that only ICSI was used as the insemination technique for women undergoing PGT-M contributed to minimize the effect of paternal factors.

**WIDER IMPLICATIONS OF THE FINDINGS:**

Ovarian stimulation and response to stimulation did not affect blastocyst euploidy rates in women undergoing PGT-M cycles. However, in women aged 31–40 years old, there was a significant decline in blastulation rates as the number of retrieved oocytes increased.

**STUDY FUNDING/COMPETING INTEREST(S):**

This study was supported by the National Natural Science Foundation of China (Grant No. 81701407, 82301826); the National Key Research and Development Program of China (2022YFC2702901, 2022YFC2703004); China Postdoctoral Science Foundation (2022M710261), and China Postdoctoral Innovation Talent Support Program (BX20220020). There is no conflict of interest.

**TRIAL REGISTRATION NUMBER:**

N/A.

## Introduction

WHAT DOES THIS MEAN FOR PATIENTS? This study looks at whether the medical interventions, i.e. ovarian stimulation, used for developing multiple oocytes for IVF and embryo transfer cycles have effects on embryo euploidy. Preimplantation genetic testing for monogenic disorders (PGT-M) is a process primarily used to screen embryos before they are transferred for maternally or paternally derived single gene disorders; however, the process simultaneously analyses the number and integrity of chromosomes, i.e. the euploidy status of the embryos. Patients who undergo PGT-M typically have no additional risks of aneuploidy (missing or extra chromosomes), which means they are an ideal study population for exploring the effects of ovarian stimulation on embryo euploidy. In this study, a large cohort of patients undergoing PGT-M cycles were included. The researchers concluded that ovarian stimulation, including different protocols, different hormone dosages, different intervention durations, and different types of hormones, had no effect on rates of euploidy in the embryos.

Aneuploidy, which refers to chromosomal copy-number abnormalities, affects around a half of human preimplantation embryos (Rubio *et al.*, 2019). It has been reported to be the leading cause of recurrent implantation failure, embryo developmental arrest, pregnancy loss, and congenital disorders. The mechanism causing aneuploidy is either a meiotic error during gametogenesis and fertilization with an unbalanced gamete (leading to uniform aneuploidy) or a mitotic error during embryo development (resulting in the coexistence of euploid and aneuploid cells, i.e. mosaicism). While maternal age has long been acknowledged as a crucial factor affecting euploidy rates, fertility centers and physicians can achieve varied euploidy rates even among young oocyte donors (Munne *et al.*, 2017; McCulloh *et al.*, 2019). This finding leads to speculation that medical interventions may also have potential effects on the euploidy status.

Ovarian stimulation in IVF–embryo transfer is performed with the objective of obtaining a specific number of oocytes that yield available euploid embryos. Animal research has shown that superovulation by administering a high gonadotropin dose has detrimental effects on oocyte development and embryo chromosome abnormalities (Lee *et al.*, 2005; Roberts *et al.*, 2005). However, human studies investigating the effects of ovarian stimulation on embryo euploidy in ART cycles have shown conflicting results. Several studies have found that the incidence of aneuploidy is affected by exogenous gonadotropin dosage (Baart *et al.*, 2007), ovarian stimulation protocols (Wang *et al.*, 2022), stimulation durations (Cascales *et al.*, 2021), and the number of oocytes retrieved (Haaf *et al.*, 2009), while others have reported no such effects (Barash *et al.*, 2017; La Marca *et al.*, 2017; Wu *et al.*, 2018; Hong *et al.*, 2019; Irani *et al.*, 2020).

The presence of heterogeneity within the study population provides a plausible explanation for the observed variations. In the majority of cases, the study population consisted mainly of patients who underwent preimplantation genetic testing for aneuploidy (PGT-A) to obtain the euploidy status of the embryos. However, PGT-A is carried out following heterogeneous indications, including recurrent miscarriage, recurrent implantation failure, advanced age, and severe teratozoospermia, while some patients are only desiring information regarding the health status of their embryos, resulting in differential risks of aneuploidy among this population (La Marca *et al.*, 2017). A recent study used patients who underwent PGT for structural chromosome rearrangements (PGT-SR), which is for couples with known chromosome rearrangements, as the study group used only the *de novo* chromosomal abnormality rate as the outcome measure (Liu *et al.*, 2022). However, it is unknown whether and to what extent intrinsic chromosomal abnormality influences gametes and embryos. Several studies have included oocyte donors as the study population; however, the sample sizes have been notably limited (Rubio *et al.*, 2010; Labarta *et al.*, 2012).

Another important consideration is the stage of embryo development at which the biopsy is conducted. Generally, the biopsy for euploidy assessment is performed on either cleavage-stage embryo on Day 3 or blastocysts on Days 5–6. Studies utilizing cleavage-stage biopsies are prone to find that more robust ovarian stimulation or higher responses to stimulation are associated with higher aneuploidy rates (Baart *et al.*, 2007; Haaf *et al.*, 2009). On the contrary, most of the studies utilizing trophectoderm (TE) biopsies of blastocysts have shown that ovarian stimulation and oocyte yield do not affect blastocyst euploidy rates (Barash *et al.*, 2017; La Marca *et al.*, 2017; Wu *et al.*, 2018; Hong *et al.*, 2019; Irani *et al.*, 2020). One hypothesis is that higher ovarian stimulation and responses indeed increase the rates of cleavage-stage embryo aneuploidy, but aneuploid cleavage-stage embryos cannot develop to usable blastocysts and are thus eliminated from the analyses involving blastocyst biopsy. Another hypothesis is that aneuploid cleavage-stage embryos can deplete their aneuploid cells and develop to euploid blastocysts (Yang *et al.*, 2021). Therefore, it is important to investigate the effects of ovarian stimulation and responses on the quality of oocytes and embryos before the blastocyst stage, such as through their rates of fertilization and blastulation.

To mitigate the potential influence of selection bias, we explored this controversial issue in a large cohort of patients undergoing preimplantation genetic testing for monogenic disorders (PGT-M). PGT-M is primarily used to screen for maternally or paternally derived monogenic variants, but the euploidy status of embryo is detected simultaneously. Compared with patients undergoing PGT-A and PGT-SR, those who undergo PGT-M typically have no additional risks of aneuploidy (except possibly for advanced age, which typically be solved by age-based subgroups), thus providing a better study population for evaluating the effect of ovarian stimulation on euploidy rates. Moreover, the effects of ovarian stimulation on the fertilization and blastulation rates in the PGT-M cycles were explored.

## Materials and methods

### Study subjects

All clinical data were collected from the Reproductive Center of Peking University Third Hospital, Beijing, China. All PGT-M cycles in which couples had natural conception of at least one child with a specific monogenic disorder and were not diagnosed with infertility were screened for eligibility between June 2014 and July 2021. Patients who were at increased *a priori* risks for chromosomally abnormal embryos, including abnormal karyotype carriers, recurrent miscarriage, recurrent implantation failure, and severe teratozoospermia, were excluded. To account for the impact of advanced maternal age, we included different age subgroups among the women participating in the study. Diminished ovarian reserve (DOR) has been shown to affect euploidy rates (Jaswa *et al.*, 2021; La Marca *et al.*, 2022); thus, patients with DOR were also excluded from the statistical analysis. DOR was diagnosed according to anti-Müllerian hormone (AMH) <1.1 ng/ml, antral follicle count (AFC) <5–7, or basal FSH ≥10 IU/L (Ferraretti *et al.*, 2011; Tal and Seifer, 2017). This study was approved by the Peking University Third Hospital Medical Science Research Ethics Committee.

### Clinical treatments

The ovarian stimulation regimen, the trigger of final oocyte maturation, oocyte retrieval, and fertilization were carried out according to standardized protocols described previously (Wang *et al.*, 2017). Patients were offered four stimulation protocols, including the antagonist protocol, long-term protocol, ultra long-term protocol, and short-term protocol. The ultra long- and long-term protocols include a long-acting GnRH agonist (GnRH-a) 2.5–3.75 mg on the first or second day of their menstrual cycle or 1.25–1.8 mg on the Day 1 week before the expected day of menstruation, respectively. Following downregulation, ovarian stimulation was initiated using recombinant gonadotropins. The short-term protocol involved the simultaneous administration of a short-acting GnRH-a and recombinant gonadotropins from the second to the third day of the menstrual cycle to stimulate an ovarian response. In patients treated with the GnRH-antagonist protocol, recombinant gonadotropins were initiated on the second day of the menstrual cycle, and treatment with a GnRH antagonist was initiated when at least one follicle reached 12 mm in diameter and was continued until the day HCG was administered. Recombinant HCG at a dose of 250 μg was administered to trigger oocyte maturation when two or more follicles measured a diameter ≥18 mm or three or more follicles measured ≥17mm, and oocyte retrieval was performed 36 h later.

### Laboratory protocols

Oocytes were inseminated ∼4–6 h after oocyte retrieval. Only ICSI was used as an insemination technique for women undergoing PGT-M. Only oocytes with the first polar body extruded (metaphase II, MII) were subjected to ICSI. Assessment of fertilization was carried out ∼16–18 h after insemination and was evaluated by the presence of two pronuclei. Embryo qualities were assessed 68–72 h after insemination according to the criteria established by the Istanbul consensus workshop on embryo assessment. All embryos were used for blastocyst culture.

Embryos were biopsied on Day 5 or 6 based on the time of blastulation. The evaluation of blastocyst morphology was conducted according to the Gardner classification (Gardner and Schoolcraft, 1999). Blastocysts with good grades (AA/AB/BA) and fair grades (BB) were selected for biopsy. In cases where no good or fair quality blastocysts were available, borderline fair quality blastocysts (BC/CB) were considered for biopsy. Blastocysts of poor quality (CC) were excluded and not subjected to further analysis. After immobilizing the embryo, the zona pellucida was perforated with laser pulses, and a biopsy pipette of 20-μm internal diameter was used to aspirate 5–10 cells for PGT-M analysis.

### PGT-M analysis

PGT-M was performed using a next-generation sequencing (NGS)-based PGT procedure as previously described (Yan *et al.*, 2015). This method, termed ‘mutated allele revealed by sequencing with aneuploidy and linkage analyses’ (MARSALA), can simultaneously detect a single-gene disorder and aneuploidy and enables linkage analysis. Aneuploidy is determined by copy number variations, whereas single-nucleotide variations associated with monogenic diseases are detected by PCR amplification of the multiple annealing and looping-based amplification cycles (MALBAC) products. Embryos with <30% aneuploid cells were considered euploid, those with 30–70% aneuploidy were classified as mosaic and those with >70% were considered aneuploid.

### Outcome measures

IVF cycles were divided into five subgroups according to maternal age: <30, 31–34, 35–37, 38–40 and >40 years old (y.o.). The primary outcome was the euploidy rate, which was calculated by dividing the number of euploid embryos, with 46 chromosomes, by the total number of biopsied embryos. Secondary outcomes included the normal fertilization rate (numbers of 2PN embryos/MII oocytes) and the blastulation rate (numbers of blastocysts/2PN embryos).

### Statistical analysis

Continuous variables were tested for normality and expressed as means (SD) or medians (Q1, Q3). Statistical significance was evaluated using the ANOVA or a non-parametric test to compare continuous variables. The correlation between statistically significant parameters and outcome measures was investigated utilizing generalized linear regression as appropriate. Statistical analysis was performed using SPSS v.24.0 (IBM, Armonk, NY, USA). Significance was set at *P* < 0.05 for two-tailed tests. Data visualization was executed using R statistical software, version 4.1.1 (http://www.R-project.org/).

## Results

A total of 642 patients undergoing PGT-M were screened, and 17 patients were excluded for carrying abnormal karyotypes, while 8 were excluded for a history of recurrent miscarriage. Consequently, 617 women (aged between 22 and 45 y.o.) with 867 PGT-M cycles involving 12 874 retrieved oocytes and 3106 biopsied blastocysts were included in the study. The demographic parameters and clinical characteristics are presented in Table 1. The mean BMI increased steadily with graded age groups (*P* < 0.001). Median serum AMH declined gradually with age groups, from 3.25 ng/ml in women younger than 30 y.o. to 0.8 ng/ml in women older than 40 (*P* < 0.001). Accordingly, basal FSH measured on menstrual day 2 increased with women’s age (*P* = 0.003). A total of 33 patients were diagnosed with DOR. The prevalence of DOR was 1.6% (3/189) in women younger than 30 y.o., 3.0% (8/268) in women aged 30–34 y.o., 5.5% (5/91) in women aged 35–37 y.o., 13.7% (7/51) in women aged 38–40 y.o., and 55.6% (10/18) in women older than 40 y.o. These women were excluded from the latter statistical analyses.

**Table 1:** Maternal characteristics of 867 PGT-M cycles.

Parameters	Age (#cycles)					*P* value
	<30 (n = 240)	30–34 (n = 375)	35–37 (n = 146)	38–40 (n = 83)	>40 (n = 23)	
Age (years)	27.07 ± 1.71	31.95 ± 1.39	35.82 ± 0.80	38.94 ± 0.89	41.87 ± 1.10	–
BMI (kg/m^2^)	21.73 ± 3.25	22.32 ± 3.07	22.56 ± 2.73	23.40 ± 3.86	23.81 ± 2.53	<0.001
AMH (ng/ml) [M (Q1, Q3)]	3.25 (2.12, 5.29)	3.18 (1.79, 4.76)	2.55 (1.39, 4.10)	1.60 (1.01, 3.05)	0.80 (0.45, 1.59)	<0.001
Basal FSH (mIU/ml) [M (Q1, Q3)]	6.14 (4.92, 7.44)	6.51 (5.41, 7.97)	7.02 (5.63, 8.23)	7.08 (5.86, 9.17)	7.46 (5.95, 9.93)	0.003
Total gonadotropin dosage (IU) [M (Q1, Q3)]	1812.5 (1425, 2412.5)	2175 (1725, 2850)	2475 (1875, 3037.5)	2925 (2250, 3787.5)	2850 (2550, 3650)	<0.001
Gonadotropin duration (days) [M (Q1, Q3)]	10 (9, 11)	10 (9, 12)	10 (9, 11)	10.5 (9, 12)	10 (9, 11.5)	0.05
Oocytes retrieved [M (Q1, Q3)]	16 (11, 21)	14 (9, 20)	12 (8, 17)	10 (7, 15)	7 (4, 9.5)	<0.001
MII oocytes [M (Q1, Q3)]	12 (9, 17)	11 (7.25, 15)	9 (6.75, 14)	9 (5.75, 12)	7 (3.5, 8.5)	<0.001
Embryos (2PN) obtained [M (Q1, Q3)]	9 (6, 13)	8 (5, 12)	7 (4, 11)	6 (4, 9)	5 (2.5, 7)	<0.001
Embryos biopsied [M (Q1, Q3)]	3 (2, 6)	3 (2, 5)	3 (1, 4)	2 (1, 4)	1 (1, 3)	<0.001
Euploid embryos [M (Q1, Q3)]	2 (1, 4)	2 (1, 3)	1 (0, 3)	1 (0, 2)	0 (0, 1)	<0.001
Aneuploid embryos [M (Q1, Q3)]	1 (0, 1)	0 (0, 1)	1(0, 1)	1 (0, 2)	1 (1, 2)	<0.001
Whole chromosome	0 (0, 1)	0 (0, 1)	1 (0, 1)	1 (0, 2)	1 (1, 2)	<0.001
Segmental chromosome	0 (0, 1)	0 (0, 1)	0 (0, 0)	0 (0, 0)	0 (0, 0)	0.164
Mosaic embryos [M (Q1, Q3)]	1 (0, 1)	0 (0, 1)	0 (0, 1)	0 (0, 1)	0 (0, 0.5)	0.010
MII rates [%, M (Q1, Q3)]	80 (69.23, 88.89)	83.33 (70.65, 90.91)	83.33 (70, 93.33)	83.33 (74.39, 96.18)	88.89 (87.5, 90.91)	0.121
Fertilization rates [%, M (Q1, Q3)]	73.68 (62.5, 83.33)	76.57 (65, 86.67)	77.78 (60, 87.29)	78.42 (66.67, 87.05)	75 (70, 85.71)	0.345
Blastulation rates [%, M (Q1, Q3)]	41.67 (28.57, 60)	40 (25, 55.56)	40 (25, 63.35)	40 (25, 59.58)	30 (16.67, 50)	0.633
Euploidy rates [%, M (Q1, Q3)]	66.67 (50, 100)	66.67 (50, 100)	50 (0, 75)	50 (0, 100)	0 (0, 37.5)	<0.001
Aneuploidy rates [%, M (Q1, Q3)]	11.11 (0, 33.33)	0 (0, 33.33)	25 (0, 50)	33.33 (0, 66.67)	100 (41.67, 100)	<0.001
Whole chromosome	0 (0, 16.67)	0 (0, 20)	18.33 (0, 50)	33.33 (0, 51.39)	100 (50, 100)	<0.001
Segmental chromosome	0 (0, 12.5)	0 (0, 10.83)	0 (0, 0)	0 (0, 0)	0 (0, 0)	0.209
Mosaic rates [%, M (Q1, Q3)]	14.29 (0, 33.33)	0 (0, 25)	0 (0, 33.33)	0 (0, 33.33)	0 (0, 12.5)	0.106
Cycles with at least one euploid embryo (%)	90.8	88.5	74.0	73.5	30.4	–

AMH, anti-Müllerian hormone; PN, pronuclear; M, median; Q1, first quartile; Q3; third quartile; –, not applicable.

The most commonly used ovarian stimulation protocol was the GnRH antagonist protocol (635 cycles, 76.5%), followed by the GnRH-agonist long-protocol (153 cycles, 18.4%), ultra-long GnRH-agonist protocol (29 cycles, 3.5%), and the short protocol (13 cycles, 1.6%). Women aged ≥38 years exhibited a significantly higher total gonadotropin dosage compared to those younger than 35 years (*P* < 0.001). However, the duration of stimulation did not demonstrate a statistically significant difference across the five age groups (*P* = 0.05) (Table 1). The response to stimulation, as assessed by the number of oocytes retrieved, declined with age, from a median number of 16 in women younger than 30 y.o. to 7 in women older than 40 (*P* < 0.001). Subsequently, the numbers of MII oocytes, 2PN embryos, available blastocyst for biopsy, and euploid embryos all reduced with age (*P* < 0.001). However, the oocyte maturation (MII oocytes) rates, normal fertilization (2PN) rates, and blastulation rates were comparable among different age groups (*P* = 0.121, 0.345, and 0.633, respectively). Only euploidy rates declined with age (*P* < 0.001). Median euploidy rates were in the range of 60–70% in patients younger than 35 y.o. and sharply declined in patients older than 35 y.o. The prevalence of cycles in which at least one euploid embryo was obtained also decreased with age, from the highest (90.8%) in women younger than 30 y.o. to the lowest (30.4%) in women older than 40 y.o. (Table 1).

**Figure 1.**
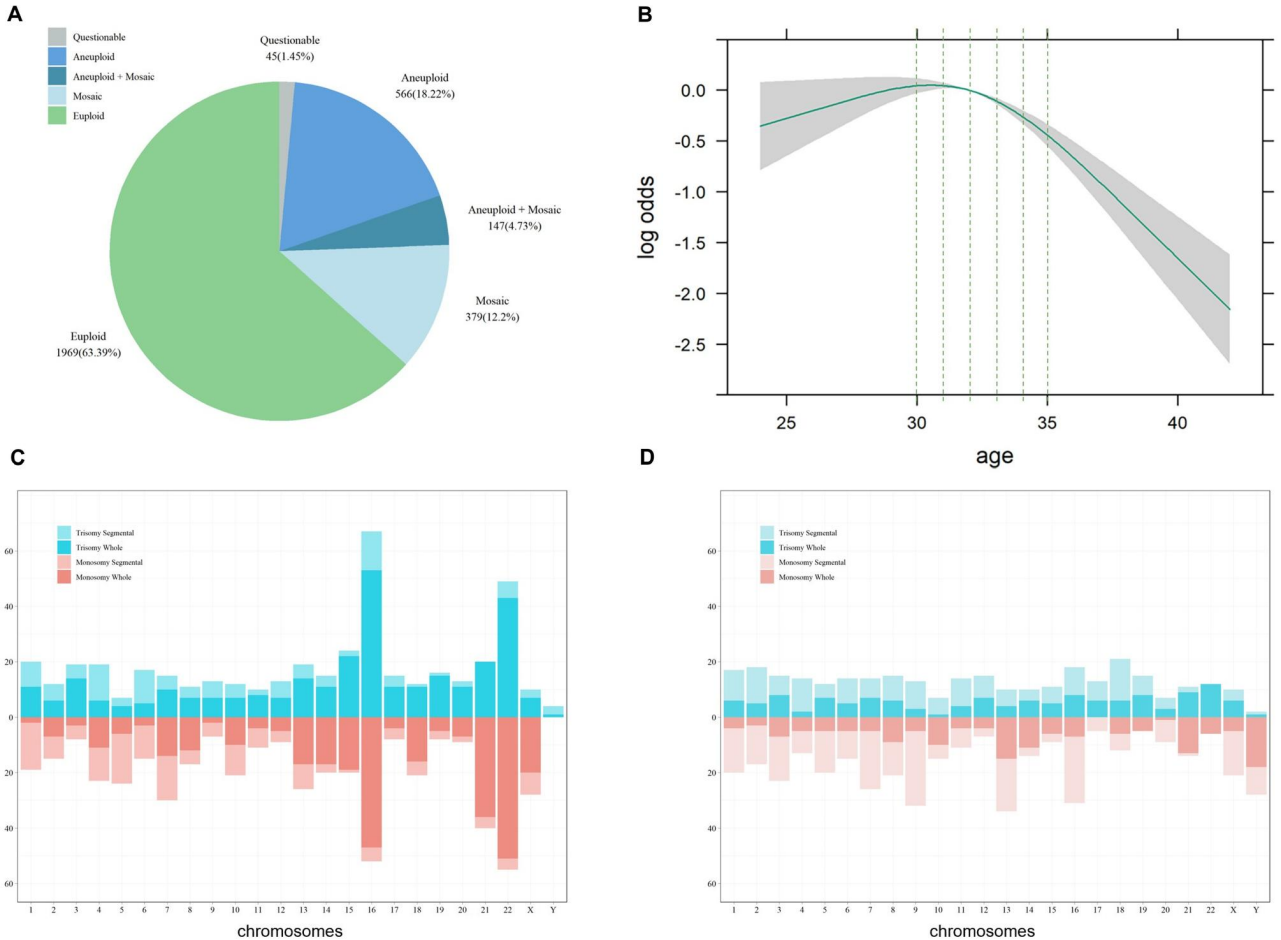
**An overview of the euploidy status of 3106 blastocysts**. (**A**) The incidence of euploidy, aneuploidy, and mosaicism after trophectoderm biopsy. (**B**) The association between maternal age and blastocyst euploidy rates. (**C**) Distribution of aneuploidy rates and types of aneuploidy across the chromosomes. (**D**) Distribution of mosaicism rates and types of mosaicism across the chromosomes.

An overview of the blastocyst euploidy status is presented in Fig. 1. Among the 3106 biopsied embryos, 1969 (63.4%) were euploid, 566 (18.2%) were aneuploid, 379 (12.2%) were mosaic, and 147 (4.7%) were both aneuploid and mosaic (Fig. 1A). The association between maternal age and blastocyst euploidy rates is shown in Fig. 1B. The euploid rate declined from the age of 32 y.o. and significantly declined after the age of 34 y.o. The distribution of aneuploid and mosaic chromosomes is presented in Fig. 1C and D, respectively. Aneuploidy most frequently occurred in chromosome 16 and chromosome 22. However, there were no dominant chromosomes for mosaicism.

After excluding patients with DOR, 830 PGT-M cycles were analyzed to investigate the effect of ovarian stimulation on embryo euploidy rates, normal fertilization rates, and blastulation rates, respectively. Different ovarian stimulation parameters (including different protocols, different starting and total gonadotropin dosages, different stimulation durations, and whether HMG supplementation was used) were all associated with comparable euploidy rate in all age groups (all *P* > 0.05) (Table 2). The response to stimulation, as reflected by the number of oocytes retrieved, had no effect on euploidy rates in all age groups (all *P* > 0.05) (Table 2). Notably, the sample size in the older than 40 y.o. group was too small, leading to quite low statistical power. The association between total gonadotropin dosage with euploidy rates for the five age groups is presented in Supplementary Fig. S1.

**Table 2:** Median euploidy rates (%) of blastocysts for women of different age groups.

Parameters	Age (#cycles)				
	<30 (n = 237)	31–34 (n = 365)	35–37 (n = 139)	38–40 (n = 76)	>40 (n = 13)
Stimulation protocol					
GnRH antagonist	66.67 (50, 87.85)	66.67 (50, 100)	50 (18.75, 75)	50 (25, 66.67)	0 (0, 0)
Long-protocol	71.43 (45, 100)	80 (50, 100)	58.33 (18.75, 100)	33.33 (0, 75)	25 (12.5, 70.83)
Ultra-long protocol	83.33 (36.67, 100)	63.33 (0, 100)	70.83 (16.67, 93.75)	100 (25, 100)	–
Short protocol	100 (50, –)	58.33 (12.5, 91.67)	66.67 (0, –)	50 (33.33, –)	–
*P* value	0.514	0.560	0.865	0.331	0.055
Starting gonadotropin dosage (IU)					
≤150	66.67 (50, 100)	66.67 (50, 100)	61.91 (0, 81.25)	45.24 (0, 75)	–
>150∼<300	66.67 (46.43, 100)	66.67 (50, 100)	50 (0, 75)	50 (33.33, 100)	–
≥300	66.67 (33.33, 100)	80 (50, 100)	57.14 (33.33, 80)	41.67 (0, 100)	12.5 (0, 68.75)
*P* value	0.749	0.358	0.691	0.387	–
HMG					
No	66.67 (50, 100)	66.67 (50, 100)	57.14 (25, 75)	50 (25, 75)	0 (0, 0)
Yes	60 (36.67, 100)	80 (50, 100)	50 (18.75, 80)	50 (20, 100)	25 (0, 70.83)
*P* value	0.631	0.410	0.886	0.770	0.217
Total gonadotropin dosage (IU)					
<2500	66.67 (50, 100)	66.67 (50, 100)	60 (0, 80)	50 (33.33, 66.67)	
2500–<4000	66.67 (50, 100)	66.67 (50, 100)	50 (26.79, 75)	50 (23.61, 100)	0 (0, 75)
≥4000	75 (21.43, 80)	66.67 (0, 100)	33.33 (0, 75)	33.33 (0, 50)	25 (0, –)
*P* value	0.459	0.841	0.582	0.409	0.675
Gonadotropin duration (day)					
<10	66.67 (50, 87.85)	75 (45, 100)	63.33 (31.25, 100)	33.33 (0, 50)	50 (0, –)
10–12	66.67 (45, 100)	66.67 (50, 100)	50 (0, 75)	50 (31.25, 100)	0 (0, 56.25)
>12	66.67 (60, 80)	64.58 (0, 100)	66.67 (0, 100)	33.33 (21.11, 100)	–
*P* value	0.541	0.381	0.407	0.201	0.503
Oocytes retrieved					
<10	60 (0, 100)	80 (50, 100)	50 (0, 100)	33.33 (0, 58.33)	0 (0, 81.25)
10–19	66.67 (50, 100)	66.67 (50, 100)	58.57 (0, 76.25)	50 (26.79, 100)	0 (0, 25)
≥20	66.67 (50, 82.58)	66.67 (50, 86.16)	50 (34.38, 72.92)	45.24 (31.25, 100)	–
*P* value	0.277	0.395	0.912	0.218	0.870

HMG: human menopausal gonadotrophin; –, not applicable.

Euploidy rates were calculated as median (M) and inter-quartile ranges (Q1, Q3) for numbers of euploid blastocysts divided by the total number of blastocysts in each cycle.

**Figure 2.**
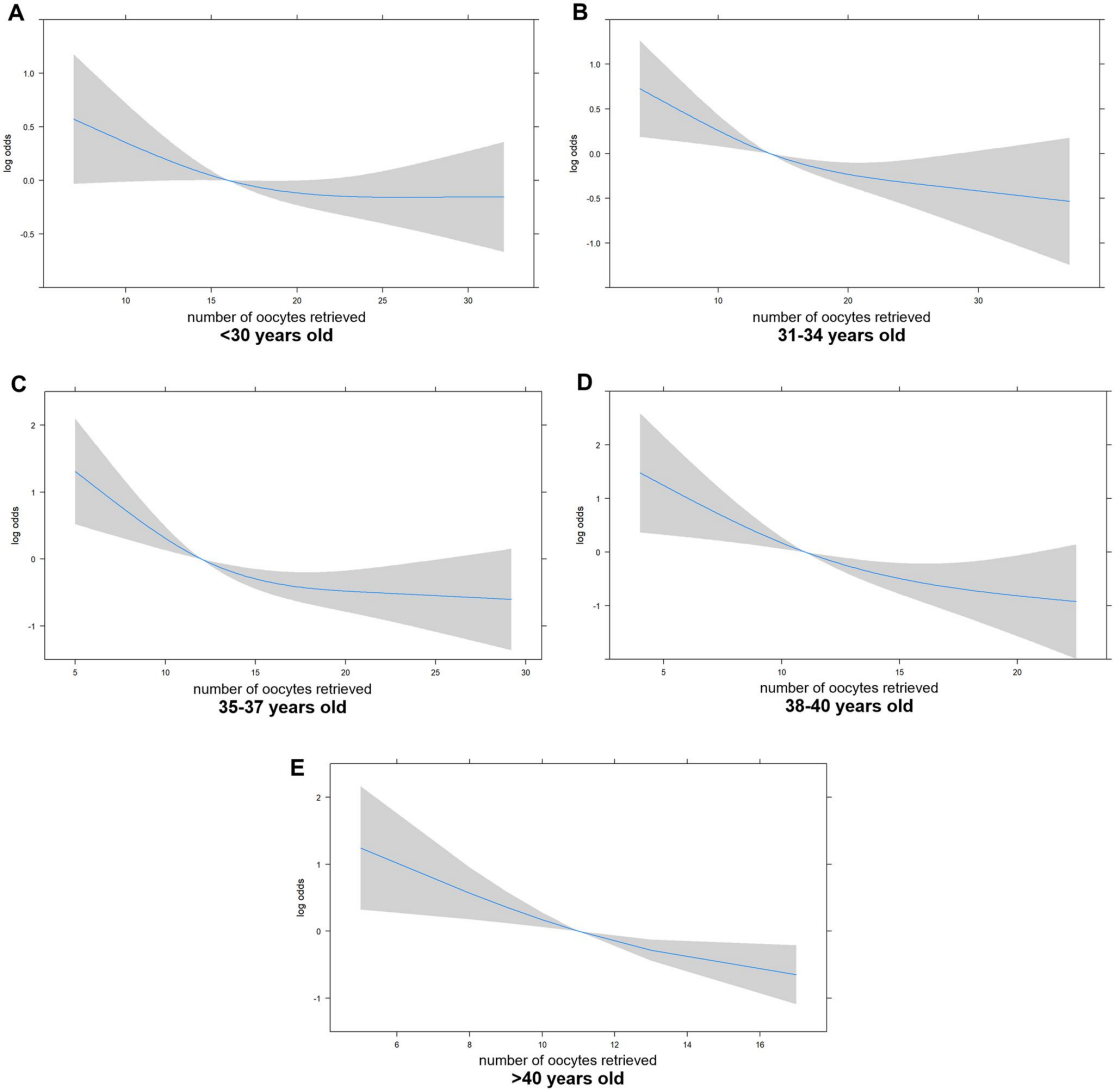
**The association between embryo blastulation rates and the number of oocytes retrieved for five age groups of women**. Blastulation rates were calculated as median (M) and inter-quartile ranges (Q1, Q3) for numbers of blastocysts divided by the number of two pronuclear (2PN) embryos in each cycle.

All ovarian stimulation parameters were associated with comparable fertilization rates in all age groups (all *P* > 0.05) (Supplementary Table S1). Some parameters of ovarian stimulation influenced the blastulation rate in certain age groups (Supplementary Table S2). In the 31–34 y.o. group, the stimulation protocol, starting and total gonadotropin dosage, HMG supplementation, and number of oocytes retrieved were found to affect blastulation rates (*P* < 0.05). After linear regression entering the above parameters with *P *<* *0.05, the starting gonadotropin dosage and numbers of oocytes retrieved remained statistically significant. Intriguingly, the starting dose had contrary effects on blastulation rates in different age groups: blastulation rates declined with the starting dose in women aged 31–34 y.o. (*P* = 0.005) but increased with the starting dose in women aged 35–37 y.o. (*P* = 0.017). The trend in blastulation rates declined gradually with an increase in the number of oocytes retrieved (Fig. 2), with statistical significance in women aged 31–34, 35–37, and 38–40 y.o. (*P* = 0.001, <0.001, and 0.012, respectively) (Supplementary Table S2).

## Discussion

We evaluated the effect of ovarian stimulation on embryo euploidy in women undergoing PGT-M. It was found that parameters associated with ovarian stimulation, such as increased gonadotropin dosage and prolonged stimulation duration, and numbers of retrieved oocytes had no significant effects on the blastocyst euploidy rate. However, specific parameters related to ovarian stimulation and ovarian response were found to impact blastulation rates in certain age groups. The most significant finding is that blastulation rates significantly declined with an increase in the number of oocytes retrieved in women aged 31–40 y.o.

Whether ovarian stimulation affects embryo euploidy has not been well-explored. A systematic review encompassing 15 studies published from 2007 to 2019 (Rodriguez-Purata *et al.*, 2022), along with more recent investigations (Hong *et al.*, 2019; Irani *et al.*, 2020; Cascales *et al.*, 2021; Liu *et al.*, 2022), yielded inconclusive findings due to several factors: (i) variations in the study population, (ii) biopsies performed at different stages of embryo development, and (iii) evolution of platforms utilized for ploidy assessment. Irani *et al.* (2020) reviewed 2230 PGT-A cycles and used array comparative genomic hybridization (aCGH)/NGS to analyze the euploidy rates of the blastocysts. The results indicated that neither ovarian stimulation nor the response to stimulation influenced the euploidy status. The same conclusion was reached in similar studies, which analyzed the effect of ovarian stimulation on blastocyst euploidy in patients who underwent PGT-A using aCGH/SNP despite different levels of gonadotropin dosages used (Barash *et al.*, 2017; La Marca *et al.*, 2017; Wu *et al.*, 2018). Hong *et al.* (2019) retrospectively compared blastocyst aneuploidy rates in 369 patients with natural cycles and 2846 patients undergoing ovarian stimulation and concluded that blastocyst aneuploidy rates were not impacted by follicular stimulation with exogenous gonadotropins. In contrast, Baart *et al.* (2007) conducted a randomized controlled trial involving 111 women who did not exhibit a pre-existing increased risk for chromosomally abnormal embryos. The study aimed to compare the euploidy rates of cleavage-stage embryos generated through a mild stimulation regimen with those obtained via a conventional high-dose exogenous gonadotropin regimen. The analysis employed fluorescence *in situ* hybridization to assess the chromosomal status of the embryos (the copy number of 10 pairs of chromosomes were determined). Compared with the mild stimulation regimen, they found that the high-dose gonadotropin regimen resulted in lower euploidy rates. Haaf *et al.* (2009) performed polar body analysis using FISH to indirectly reveal the embryo euploidy status and found that a high yield of oocytes after ovarian stimulation correlated with a high chromosome error rate in women up to 40 years.

Analyses of studies on this topic revealed that investigations that performed euploidy analysis at the blastocyst stage tended to conclude that there was no correlation between ovarian stimulation and euploidy rates regardless of the study population. Similarly, our study found that ovarian stimulation and the response to stimulation did not affect blastocyst euploidy rates in patients undergoing PGT-M. This finding is important owing to the fact that patients undergoing PGT-M do not have an increased risk for chromosomally abnormal embryos, unlike patients undergoing PGT-A or PGT-SR. As a result, this subset of patients provides more compelling evidence regarding the correlation between ovarian stimulation and euploidy rates.

TE biopsies at the blastocyst stage provide more precise euploidy status than cleavage-stage biopsies because of the chromosomal instability in early human embryos (Yang *et al.*, 2021). However, only usable blastocysts were biopsied. The embryos that did not develop to blastocyst stage, possibly due to chromosomal abnormalities, were not counted. Therefore, it is necessary to investigate the effect of ovarian stimulation on fertilization and blastulation rates, which reveal the oocyte competence before the blastocyst stage. In this study, we found that blastulation rates significantly declined as the number of retrieved oocytes increased in women aged 31–40 y.o., which is consistent with the study by Rubio *et al.* (2010) who found a significant increase in blastocyst development rates in high responder donors after receiving a reduced dose of gonadotrophins in a second cycle. Considering the study by Baart *et al.* (2007), which demonstrated a negative correlation between higher gonadotropin dosage and euploidy rates in cleavage-stage embryos, as well as the research by Haaf *et al.* (2009) indicating that a greater number of oocytes obtained is associated with an increased likelihood of chromosome errors in early-stage embryos (around 8–10 h following sperm injection), a plausible hypothesis emerges. This hypothesis suggests that excessive ovarian response may contribute to chromosomal abnormalities in the early stages of embryo development, thereby hindering their progression to the blastocyst stage. Consequently, only embryos that successfully reach the blastocyst stage are selected for TE biopsies, thereby leaving the blastocyst euploidy rates unaffected.

Although the tendency that blastulation rates declined with an increase in the number of oocytes retrieved was also found in women aged <30 or >40 y.o., it did not reach statistical significance. The possible explanation is that maternal age plays a dominant role in the two age groups. In women aged <30 y.o., maternal age influences the achievement of high euploid rates. This dominant effect is strong enough to mitigate the influence of other factors. This hypothesis can also explain the findings of Labarta *et al.* (2012), who reported that the euploid rate of cleavage-stage embryos in young oocyte donors (mean age 25.4 y.o.) was not influenced by ovarian stimulation compared with unstimulated cycles. However, in women aged over 40 y.o., the maternal age is a detrimentally dominant effect for overall low euploid rates, meaning that the influence of other factors was concealed. This hypothesis was supported by the finding of Haaf *et al.* (2009), who reported that a high oocyte yield is associated with an increased chromosome error rate in women aged ≤40 y.o. but not in women aged >40 y.o.

Another interesting finding in this study is that the starting gonadotropin dosage had contrary effects on blastulation rates in different age groups. A higher starting dose was associated with a lower blastulation rate in women aged 31–34 y.o. but with a higher blastulation rate in women aged 35–37 y.o. We speculate that in younger women (31–34 y.o.), a lower starting dose is enough to recruit enough follicles, while in older women (35–37 y.o.), a higher starting dose is needed to recruit a proper number of follicles. The reason why this phenomenon was not detected in women of much younger or much older ages may also contribute to the age-dominant effect. This finding should be validated in other future studies.

The strength of this study is the homogeneity and a relatively large number of the study population. Another strength is the accuracy of the PGT platform. Limitations include its retrospective nature and a relatively small number of patients with advanced age, especially patients older than 40 y.o., leading to quite low statistical power. More prospective studies and data from patients with PGT-M are needed to confirm our findings. Second, as we considered euploid rates as outcome measures, we did not analyze the effects of ovarian stimulation on uniform aneuploidy and mosaicism, respectively. As the potential normal development of mosaic embryos is currently being investigated and is the subject of considerable debate (Treff and Marin, 2021), we will separately take uniform aneuploidy and mosaicism as outcome measures in further studies. Finally, we did not consider the effects of paternal characteristics on embryo euploid status due to the fact that blastocyst aneuploidy primarily originates from maternal meiosis (Rana *et al.*, 2023). However, sperm factors might have an effect on embryo development and the blastulation rate and therefore also the number of blastocysts analyzed. The exclusion of patients with severe teratozoospermia and the fact that only ICSI was used as an insemination technique for women undergoing PGT-M contributed to minimize the effect of paternal factors.

In conclusion, this study demonstrated that ovarian stimulation and the ovarian response to stimulation had no effect on blastocyst euploidy as well as fertilization rates in patients undergoing PGT-M. However, in women aged 31–40 y.o., there was a significant decline in blastulation rates as the number of retrieved oocytes increased, which suggests that treatment strategies for this age group should not solely focus on maximizing the yield of oocytes. Instead, there is a need for individualized regimens, particularly considering the complex relationship between maternal age and its protective or detrimental effects on embryo quality within this age range.

## Supplementary Material

hoae054_Supplementary_Data

## Data Availability

The data sets supporting the current study have not been deposited in a public repository but are available from the corresponding author on request.
