# Supplementary material for: Effects of ovarian stimulation on embryo euploidy: an analysis of 12 874 oocytes and 3106 blastocysts in cycles with preimplantation genetic testing for monogenic disorders
Source: Hum Reprod Open. 2024 Oct 3;2024(4):hoae054. doi: 10.1093/hropen/hoae054 (PMC11470209; doi:10.1093/hropen/hoae054)
Supplement: hoae054_Supplementary_Data [file hoae054_supplementary_data.docx]

**Supplementary Table S1. Median fertilization rates (%) for women of different age groups.**

| **Parameters (#cycles)** | **Age (#cycles)** | | | | |
| --- | --- | --- | --- | --- | --- |
|  | **<30 (n=237)** | **31–34 (n=365)** | **35–37 (n=139)** | **38–40 (n=76)** | **>40 (n=13)** |
| Stimulation protocol | | | | | |
| GnRH antagonist | 72.08 (62.5, 83.33) | 76.92 (66.67, 87.5) | 78.79 (60, 87.5) | 80 (66.67, 88.89) | 71.43 (60.83, 75.69) |
| long-protocol | 75 (66.67, 85.71) | 74.17 (60, 84.59) | 68.33 (54.17, 83.33) | 75 (65.15, 82.97) | 85.71 (73.33, 88.19) |
| ultra-long protocol | 86.67 (79.17, 96.43) | 79.80 (60, 89.17) | 72.22 (57.58, 83.73) | 78.26 (63.33, 81.79) | - |
| short protocol | 60 (58.82, -) | 80.35 (65.63, 91.07) | 70.59 (66.67, -) | 87.5 (66.67, -) | - |
| *P* value | 0.074 | 0.444 | 0.410 | 0.742 | 0.067 |
| Starting gonadotropin dosage (IU) | | | | | |
| ≤150 | 73.68 (63.35, 85.71) | 75 (61.43, 85.71) | 83.61 (70.24, 94.64) | 71.36 (55.30, 81.59) | - |
| ＞150~＜300 | 72.73 (60, 83.33) | 80 (66.67, 87.5) | 75.96 (57.44, 85.71) | 81.82 (70.88, 90) | - |
| ≥300 | 68.33 (60, 81.1) | 76.92 (66.18, 86.88) | 77.78 (57.14, 86.67) | 76.39 (63.35, 83.65) | 76.39 (70.24, 86.16) |
| *P* value | 0.541 | 0.265 | 0.114 | 0.145 | - |
| HMG | | | | | |
| No | 73.51 (62.5, 83.33) | 77.78 (66.67, 86.67) | 77.78 (60, 87.5) | 77.78 (66.67, 88.89) | 70.71 (70, -) |
| Yes | 70 (60, 91.88) | 75 (60.63, 87.05) | 78.18 (59.29, 87.68) | 78.57 (60, 83.33) | 77.78 (69.05, 86.61) |
| *P* value | 0.883 | 0.494 | 0.715 | 0.484 | 0.327 |
| Total gonadotropin dosage (IU) | | | | | |
| <2500 | 73.68 (62.5, 84.62) | 76.92 (66.25, 86.68) | 78.79 (60, 87.5) | 79.51 (65.63, 90.42) | - |
| 2500–<4000 | 68.75 (60, 83.33) | 75 (66.67, 86.67) | 77.78 (62.02, 86.19) | 80 (66.67, 86.94) | 75 (70, 85.71) |
| ≥4000 | 80.38 (71.25, 92.5) | 75 (60, 89.44) | 62.5 (54.55, 83.33) | 71.43 (63.64, 80) | 71.43 (66.67, -) |
| *P* value | 0.307 | 0.979 | 0.550 | 0.439 | 0.950 |
| Gonadotropin duration (day) | | | | | |
| <10 | 73.21 (62.5, 83.65) | 80 (66.67, 88.89) | 81.18 (65, 100) | 81.82 (66.67, 100) | 55.56 (33.33, -) |
| 10-12 | 71.43 (60.52, 83.33) | 76.47 (61.63, 85.71) | 75 (59.29, 85.71) | 74.04 (62.5, 82.79) | 73.21 (70.36, 84.29) |
| >12 | 75 (73.33, 85.71) | 75 (60.63, 89.72) | 66.67 (57.14, 83.33) | 80 (70.09, 88.19) | - |
| *P* value | 0.273 | 0.342 | 0.113 | 0.172 | 0.220 |
| Oocytes retrieved | | | | | |
| ＜10 | 71.43 (62.5, 87.5) | 78.89 (62.5, 100) | 77.78 (50, 100) | 80 (66.67, 93.75) | 73.21 (61.91, 86.16) |
| 10-19 | 75 (62.02, 83.97) | 75 (62.6, 85.71) | 72.73 (59.17, 85.71) | 80 (62.5, 87.30) | 77.78 (68.33, 84.44) |
| ≥20 | 73.08 (64.71, 83.33) | 78.02 (66.25, 84.44) | 80.74 (74.31, 89.11) | 76.63 (68.23, 83.20) | - |
| *P* value | 0.856 | 0.341 | 0.212 | 0.877 | 0.855 |

Fertilization rates were calculated as median (M) and inter-quartile ranges (Q1, Q3) for numbers of two pronuclear (2PN) embryos divided by the number of metaphase II (MII) oocytes in each cycle.

GnRH, Gonadotropin-releasing hormone; HMG: human menopausal gonadotrophin; IU, international unit; -, not applicable.

**Supplementary Table S2. Median blastulation rates (%) for women of different age groups.**

| **Parameters (#cycles)** | **Age (#cycles)** | | | | |
| --- | --- | --- | --- | --- | --- |
|  | **<30 (n=237)** | **31–34 (n=365)** | **35–37 (n=139)** | **38–40 (n=76)** | **>40 (n=13)** |
| Stimulation protocol | | | | | |
| GnRH antagonist | 42.86 (30, 62.26) | 42.86 (27.35, 60) | 40 (27.27, 66.67) | 37.5 (25, 58.33) | 20 (16.07, 57.14) |
| long-protocol | 38.46 (27.78, 50) | 34.85 (20, 50) | 35.42 (24.52, 60) | 44.44 (29.17, 70.83) | 50 (22.14, 58.33) |
| ultra-long protocol | 23.08 (17.69, 52.56) | 30.95 (22.32, 53.13) | 38.10 (20.83, 60.71) | 23.53 (10.10, 66.67) | - |
| short protocol | 50 (33.33, -) | 29.17 (23.56, 38.33) | 41.67 (18.18, -) | 50 (42.86, -) | - |
| *P* value | 0.120 | 0.026 (LR: 0.087) | 0.889 | 0.338 | 0.408 |
| Starting gonadotropin dosage (IU) | | | | | |
| ≤150 | 45.80 (29.85, 62.78) | 50 (31.25, 64.39) | 32.46 (17.63, 60) | 35.42 (13.49, 52.5) | - |
| ＞150~＜300 | 40 (25.66, 53.39) | 37.5 (25, 50) | 35.83 (25, 61.16) | 41.43 (21.67, 55.95) | - |
| ≥300 | 45 (27.08, 55) | 34.52 (25, 50) | 50 (28.57, 81.82) | 41.43 (27.68, 67.5) | 25 (16.07, 54.17) |
| *P* value | 0.235 | 0.002 (LR: 0.005) | 0.017 | 0.133 | - |
| HMG | | | | | |
| No | 42.48 (29.85, 60) | 40 (26.67, 60) | 37.5 (25, 62.5) | 37.5 (22.22, 53.85) | 31.43 (20, -) |
| Yes | 40 (18.33, 59.82) | 34.52 (20.56, 50) | 42.86 (28.57, 72.32) | 50 (25, 70) | 30 (15.48, 58.33) |
| *P* value | 0.488 | 0.036 (LR: 0.925) | 0.282 | 0.082 | 0.909 |
| Total gonadotropin dosage (IU) | | | | | |
| <2500 | 42.86 (29.41, 62.5) | 42.86 (27.51, 60) | 35.29 (25, 60) | 36.93 (20, 57.14) | - |
| 2500–<4000 | 38.46 (25, 50) | 38.46 (25, 50) | 42.86 (28.57, 73.21) | 40 (25, 68.33) | 42.86 (16.67, 66.67) |
| ≥4000 | 44.44 (33.33, 62.30) | 33.33 (19.09, 47.22) | 42.86 (23.08, 80) | 50 (33.33, 66.67) | 30 (20, -) |
| *P* value | 0.201 | 0.044 (LR: 0.802) | 0.238 | 0.152 | 0.357 |
| Gonadotropin duration (day) | | | | | |
| <10 | 43.65 (33.33, 62.5) | 36.36 (25.83, 58.57) | 43.91 (28.24, 63.54) | 55.56 (33.33, 66.67) | 57.14 (14.29, -) |
| 10-12 | 41.43 (26.56, 60) | 44.44 (27.27, 56.08) | 40 (25, 66.67) | 36.93 (23.20, 57.44) | 25 (17.5, 48.21) |
| >12 | 38.46 (33.33, 50) | 33.33 (18.64, 50) | 33.33 (23.08, 66.67) | 42.86 (25, 65.91) | - |
| *P* value | 0.347 | 0.085 | 0.781 | 0.265 | 0.708 |
| Oocytes retrieved | | | | | |
| ＜10 | 50 (28.57, 75) | 50 (33.33, 66.67) | 57.14 (33.33, 75) | 50 (28.57, 75) | 20 (16.07, 75) |
| 10-19 | 42.86 (29.29, 60) | 39.23 (25, 54.80) | 36.36 (25, 62.5) | 36.36 (21.11, 56.35) | 30 (0, 50) |
| ≥20 | 40 (28.57, 53.33) | 36.93 (25, 53.46) | 28.64 (20.56, 40) | 28.43 (10.97, 48.03) | - |
| *P* value | 0.096 | 0.013 (LR: 0.001) | <0.001 | 0.012 | 0.783 |

Blastulation rates were calculated as median (M) and inter-quartile ranges (Q1, Q3) for numbers of blastocysts divided by the number of two pronuclear (2PN) embryos in each cycle.

GnRH, Gonadotropin-releasing hormone; HMG: human menopausal gonadotrophin; IU, international unit; LR: linear regression; -, not applicable.


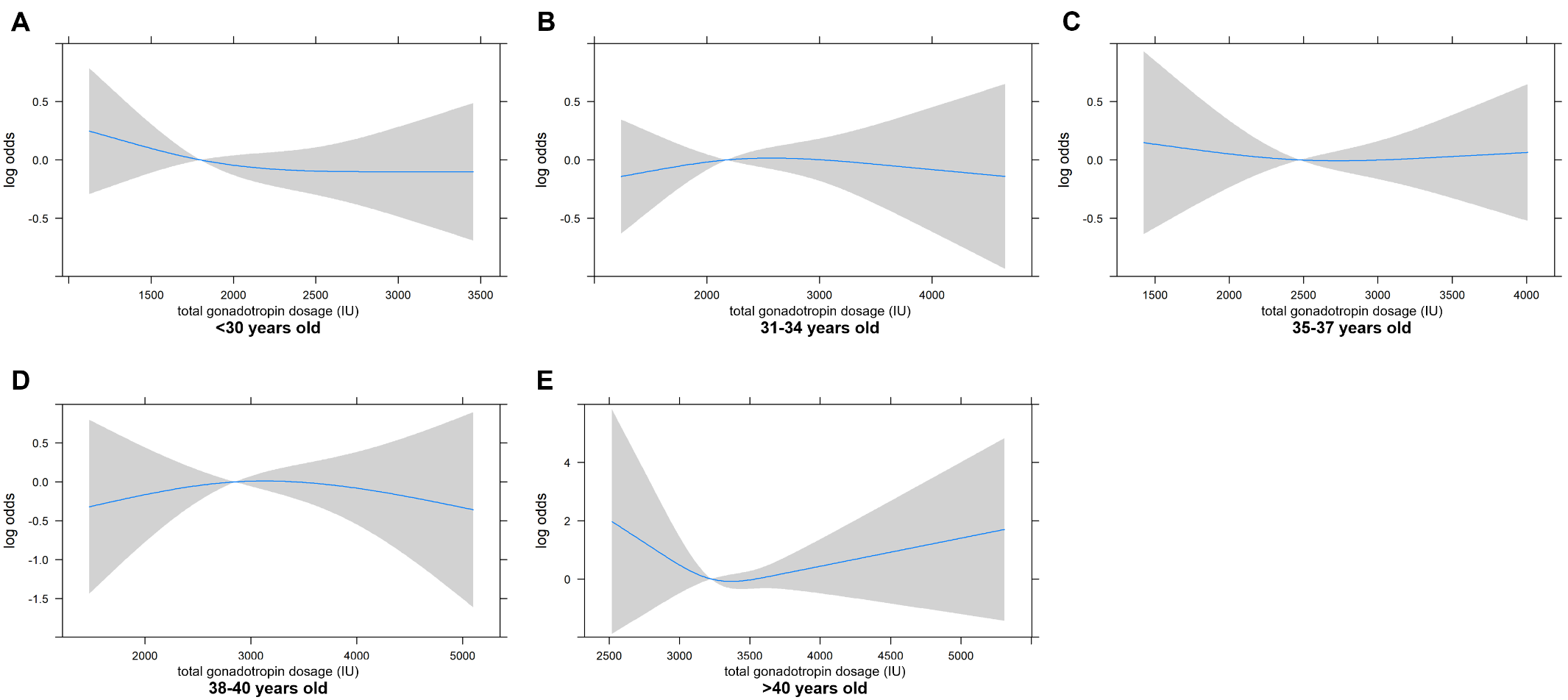


**Supplementary Figure S1** The association between median euploidy rates and total gonadotropin dosage (IU) for five age groups. Euploidy rates were calculated as median (M) and inter-quartile ranges (Q1, Q3) for numbers of euploid blastocysts divided by the total number of blastocysts in each cycle.
